# How Do Earthworms, Soil Texture and Plant Composition Affect Infiltration along an Experimental Plant Diversity Gradient in Grassland?

**DOI:** 10.1371/journal.pone.0098987

**Published:** 2014-06-11

**Authors:** Christine Fischer, Christiane Roscher, Britta Jensen, Nico Eisenhauer, Jussi Baade, Sabine Attinger, Stefan Scheu, Wolfgang W. Weisser, Jens Schumacher, Anke Hildebrandt

**Affiliations:** 1 Institute of Geosciences, Friedrich-Schiller-University Jena, Jena, Germany; 2 Max Planck Institute for Biogeochemistry, Jena, Germany; 3 UFZ, Helmholtz Centre for Environmental Research, Department of Community Ecology, Halle, Germany; 4 J. F. Blumenbach Institute of Zoology and Anthropology, Georg August University of Göttingen, Göttingen, Germany; 5 Institute of Ecology, Friedrich Schiller University Jena, Jena, Germany; 6 Department of Geography, Friedrich Schiller University Jena, Jena, Germany; 7 UFZ, Helmholtz Centre of Environmental Research, Department of Computational Hydrosystems, Leipzig, Germany; 8 Technical University Munich, Terrestrial Ecology Research Group, Department of Ecology and Ecosystem Management, Center for Food and Life Sciences Weihenstephan, Freising, Germany; 9 Institute of Stochastics, Friedrich Schiller University Jena, Jena, Germany; University of Oxford, United Kingdom

## Abstract

**Background:**

Infiltration is a key process in determining the water balance, but so far effects of earthworms, soil texture, plant species diversity and their interaction on infiltration capacity have not been studied.

**Methodology/Principal Findings:**

We measured infiltration capacity in subplots with ambient and reduced earthworm density nested in plots of different plant species (1, 4, and 16 species) and plant functional group richness and composition (1 to 4 groups; legumes, grasses, small herbs, tall herbs). In summer, earthworm presence significantly increased infiltration, whereas in fall effects of grasses and legumes on infiltration were due to plant-mediated changes in earthworm biomass. Effects of grasses and legumes on infiltration even reversed effects of texture. We propose two pathways: (i) direct, probably by modifying the pore spectrum and (ii) indirect, by enhancing or suppressing earthworm biomass, which in turn influenced infiltration capacity due to change in burrowing activity of earthworms.

**Conclusions/Significance:**

Overall, the results suggest that spatial and temporal variations in soil hydraulic properties can be explained by biotic processes, especially the presence of certain plant functional groups affecting earthworm biomass, while soil texture had no significant effect. Therefore biotic parameters should be taken into account in hydrological applications.

## Introduction

The water balance of soils is determined by the interaction of water supply and water removal due to processes such as precipitation, infiltration, run-off, percolation and evapotranspiration. For efficient soil and water management, knowledge on soil hydraulic properties, including soil hydraulic conductivity and infiltration characteristics, is necessary to understand how rainwater moves from the soil surface to the groundwater. Hydraulic conductivity describes the capacity of a porous medium to transmit water. It depends on total pore space, pore size distribution and tortuosity [1]. Soil pores are of various origin. The smallest ones (micropores) are related to the grain size distribution and constitute the largest fraction of the total pore volume [2]–[4]. Larger pores (often referred to as meso- and macropores) make up a characteristic property of the soil structure [5], [6]. Soil structure is determined by aggregates of different sizes, divided into intra-aggregate and inter-aggregate pore structures [7]. Intra-aggregate pores include micro- and mesopores, whereas inter-aggregate pores include meso- and macropores [8].

Traditionally predictions of hydraulic conductivity are based on soil texture, bulk density or organic matter content (mainly intra-aggregate pores) [9]–[11], implying a decrease in hydraulic conductivity with increasing fraction of fine grains. However, this relationship can be weakened by soil structuring processes forming larger inter-aggregate pores such as interpedal voids and biopores [3], [12]. Macropores constitute a comparatively small fraction of the total pore space, but can contribute substantially to total flow through the porous medium, mainly during high intensity rainfall events [13]–[15].

Both biotic and abiotic processes contribute to shape soil structure and aggregation of macropores. Drying and freezing causes fissures and cracks in the soil, which are prone to conduct water [16], [17]. Macropores created by both earthworms and plant roots also play a major role for preferential flow [5], [14], [18]. For example, Weiler and Naef [19] observed in grassland that the flow rate through vertically oriented macropores formed by earthworms or plant roots was higher than through the soil matrix. On the other hand, root growth can have opposite effects by clogging of pore space and thus decrease hydraulic conductivity [20].

Macropores formed by earthworms range between 2 to 11 mm in diameter [21] depending on the ecological group of earthworms, i.e. endogeic, epigeic and anecic [22]. Endogeic and epigeic earthworms that live in upper mineral soil or at the soil surface mainly form small and tortuous pores ranging between 2 and 5 mm in diameter [23]. In contrast, anecic species form pores larger than 5 mm in diameter, which may reach as deep as 2 m into the soil [24] and thus enhance infiltration into deep soil layers [25]. As a consequence of the different burrowing behaviors the impact on water flow through soil varies among the different ecological earthworm groups [18]. Further, roots form voids of different size, but the majority of pores stemming from root growth are smaller (0.1–0.6 mm) than those from earthworms [26]. However, root induced pores differ with plant species and can be much larger. For example, most of the root channels formed by the legume alfalfa were between 0.5 and 2.5 mm in diameter [27]. Besides the formation of macropores mentioned above, biotic processes are also involved in forming and stabilizing soil structure [28], [29]. Soil aggregates are more stable in biologically active soil with high carbon content, which therefore is associated with low soil bulk density and high porosity [30].

The processes contributing to structure the soil and shape its hydraulic properties are closely interlinked. For example, earthworm activity depends on a number of factors which influence soil structure and hydraulic properties, such as soil type [31] and texture [24], management practices [32] and vegetation cover [33]. Earthworms also alter above- and belowground plant productivity by forming macropores with the effect varying with plant species and functional group diversity [34], [35]. Experimental studies have shown that above- and belowground biomass production change with increasing plant diversity [36]–[38]. Furthermore, larger and longer macropores have been shown to correlate with increased plant biomass production and earthworm abundance [39]. Plant species richness does not only affect rooting density, but also improves soil stability, accumulation of organic matter and promotes the activity for soil biota [5], [40]. In addition, the presence of certain plant functional groups, such as legumes and grasses, has been shown to affect the abundance and activity of soil organisms [41], [42]. Understanding how plant species diversity, plant functional group composition and earthworms influence soil water fluxes and the resulting soil moisture distribution is important to improve predictions on how water fluxes will change in taxonomically simplified ecosystems.

Our measurements were conducted in the frame of the Jena Experiment [43], a long-term grassland biodiversity experiment with plots varying in plant species diversity and including experimental blocks differing in soil structure and texture. The design of the experiment provides the opportunity to disentangle the relative importance of soil physical and biological processes for infiltration capacity.

## Materials and Methods

### Ethics Statement

No specific permission was required for the described field studies. The field site of the Jena Experiment is a former arable land leased by the research consortium from an agricultural collective for the duration of the research grant. The land owner gave the permission for this study and field work including soil sampling and other experimental manipulations. The experiments did not involve endangered or protected species.

### Experimental Design

The study was performed on the field site of the Jena Experiment which is located in the floodplain of the Saale river near Jena (Thuringia, Germany; 50°55′N, 11°35′E, 130 m above sea level). Mean annual air temperature is 9.3°C and mean annual precipitation is 587 mm [44]. Before the establishment in 2002, the experimental field site was an arable land and highly fertilized over the last decades. After the last harvest in autumn 2000 the field was ploughed and kept fallow throughout 2001, and the experimental plant communities were established in spring 2002 [43]. The soil of the experimental site is an Eutric Fluvisol [45] developed from up to 2 m thick loamy fluvial sediments [43]. The soil texture in the upper 10 cm of the soil profile changes with increasing distance from the river gradually from sandy loam to silt clay. The sand content decreases from 40% near the river to 11% at distance, while the silt content increases proportionally from 44% to 66%. The clay content (16–23%) shows no significant spatial trend (Table S1). Plots were assembled on a 10 ha area into four blocks, arranged parallel to the river, thus accounting for changes in soil texture.

A pool of 60 native plant species common of Central European mesophilic grasslands was used to establish a gradient of plant species richness (1, 2, 4, 8, 16 and 60) and functional group richness (1, 2, 3 and 4) on 80 plots each of 20×20 m size. To account for differences in morphology and physiology, species were assigned to four functional groups: grasses (16 species), small herbs (12 species), tall herbs (20 species) and legumes (12 species). In addition to test for effects of plant species richness and functional group richness, the experimental design allows for tests caused by the presence and absence of certain functional groups and texture. Plant species richness and functional groups varied in a near-orthogonal design, because the lowest plant species richness level cannot be combined with the highest functional group number and at the 16 plant species level it was not possible to create pure legume and small-herb species mixtures. The plots were mown twice a year, and the mown material was removed from the plots shortly after cutting. All plots were weeded regularly to maintain the target species composition. More details on the experiment and management are given in Roscher et al. [43].

### Earthworm Density Manipulations

Earthworm abundance was observed and manipulated on subplots of the main experimental plots with species-richness levels of 1 (12 plots), 4 (16 plots) and 16 (14 plots) since September 2003 (Table 1). Due the unbalanced design effects of plant species richness and functional group richness are partially confounded (r = 0.438, p<0.001; Table 1). Two subplots (size 1×1 m) were located in close vicinity to each other (50 cm distance). Two treatments were established: ambient earthworm density (+ew) and earthworm density reduction (−ew). Subplots were enclosed with PVC shields (20 cm aboveground and 15 cm belowground) to decrease the re-colonization in earthworm reduction subplots [34]. Aboveground shields were removed two times a year during the mowing period. Earthworms were extracted from reduction subplots twice a year in spring (beginning of April) and autumn (end of September) by electro-shocking. A voltage was applied to the soil for 35 min *via* four octet devices [46] (DEKA 4000, Deka Gerätebau, Marsberg, Germany) powered by two 12 V batteries. During the application time the voltage was increased sequentially from 250 V (10 min) to 300 V (5 min), 400 V (5 min), 500 V (5 min) and 600 V (10 min). For more details on the arrangement of the steel rods of the octet devices and management of the earthworm subplots see Eisenhauer et al. [34]. Notably, steel rods were installed in both earthworm subplots controlling for potential side effects on infiltration. Two additional extraction campaigns on the –ew and on control subplots in 2006 (Eisenhauer, unpupl. data) confirmed that earthworm data from –ew subplots is an adequate measure of earthworm data in the +ew subplots. Extracted earthworms were identified, counted and weighted (with gut content) in the laboratory. Earthworms at the field site of the Jena Experiment mainly belong to two ecological groups [22]: anecic (*Lumbricus terrestris*) and endogeic (*Aporrectodea caliginosa, Octolasion tyrtaeum, Allolobophora chlorotica, and Aporrectodea rosea*) species. Only a small number of epigeic earthworms (*Lumbricus castaneus*) was extracted and therefore contributed to the total number and biomass of earthworms.

**Table 1:** The design of the present study in the frame of the Jena Experiment.

	Plant species richness			Plots
Plant functional group richness	1	4	16	
**1**	12	4	2	18
**2**	–	4	4	8
**3**	–	4	4	8
**4**	–	4	4	8
**Plots**	**12**	**16**	**14**	**42**

Combinations of plant species richness and plant functional group richness levels and the number of plots per diversity level for the earthworm subplots with 1, 4 and 16 plant species (n = 42, 84 subplots). Please note that this design is a selection of plots from the full design of the Jena Experiment [43]. Due to the non-orthogonal design, effects of plant species richness and functional groups richness are partially confounded (r = 0.438, p<0.001).

### Infiltration Measurement

For *in situ* infiltration measurements we used a hood infiltrometer [47] (UGT, Müncheberg, Germany). These measurements do not require preparation of the soil and therefore can be applied on an undisturbed, vegetated soil surface. In 2011, we conducted three infiltration measurement campaigns (June, September and October) on plots containing 1, 4 and 16 plant species (Table 1). The first measurement campaign was conducted at end of June, the second at the beginning of September, and the third at the end of October. We carried out paired measurements in each plot: one on the reduced and one on the ambient earthworm density subplot. The first and second measurement campaigns were conducted about 65 and 160 days after the first earthworm extraction and the third measurement campaign 30 days after the second earthworm extraction. The extracted earthworm biomass in spring was related to the infiltration capacity in June and September. The earthworm biomass from the second extraction campaign, which was conducted after the second infiltration campaign, was related to the infiltration capacity in October.

A hood with a diameter of 16 cm was placed with the open side on the undisturbed soil surface. The contact between the soil and hood was sealed with wet sand. We conducted measurements at increasingly negative matric potentials (*ψ*_M_) beginning at *ψ*_M_ = 0 m and reducing it stepwise by −0.02 m until the bubble point of the soil was reached. The bubble point refers to the matric potentials, upon which a pore channel allows for penetration of air into the hood and therefore the maximum applicable matric potential at this location. For a specific matric potential (*ψ*_M_) the equivalent diameter (*d*_e_) of the largest soil pore conducting water can be estimated after Jarvis et al. [48]. At *ψ*_M_ = 0 m the soil is saturated and the entire pore spectrum is potentially active. At smaller matrix potential larger pores are no longer active and infiltration capacity decreases. This allows evaluating infiltration capacity through different parts of the pore spectrum. At *ψ*_M_ = −0.02 m the largest active pores correspond to *d*_e_ = 1.5 mm. At each pressure level we recorded infiltration capacity until it was constant in time. This steady infiltration capacity was used for further analysis. Depending on the month most plots had a bubble point at *ψ*_M_<−0.04 m. For data analysis of individual months, we only considered infiltration capacity for *ψ*_M_ up to −0.02 m. Infiltration rates at a given matric potential are directly linked to hydraulic conductivity [49]. The flow conditions in natural soils, however, are far from ideal with anisotropic behaviour, heterogeneous initial soil water contents and flow dynamics that do not correspond to the Richards equation near soil saturation [14]. Therefore, we refrained from deriving hydraulic conductivity from our infiltration rates, for example via Wooding’s formula [49]. Instead, we worked with the observed infiltration rates, considering those as a surrogate for the capacity of the soil to conduct water at the applied matric potential.

### Soil Texture and Moisture

Soil texture was determined from soil cores at 38 locations (average of 0–100 cm depth) distributed throughout the experimental site before plot establishment (G. Büchel, pers. comm., [50]) and values for each plot were interpolated by ordinary kriging. The fraction of sand and silt are negatively correlated (clay showing no spatial trend, Table S1). Thus, in the following statistical analysis for simplicity we used the sand fraction as factor representing soil texture.

The volumetric soil water content (m^3^ m^−3^) was determined with an FDR probe (ML2x Theta Probe, Delta-T Devices, Cambridge, United Kingdom). The device was inserted from the top 6 cm deep (length of the prongs) into the soil surrounding the hood before the infiltration experiment. The average of three measurements was used for further analysis.

### Statistical Analyses

Statistical analyses were performed using the statistical software R 2.13.1 (R Development Core Team, http.//www.R-project.org). Measures of infiltration capacity were log-transformed to account for heteroscedasticity and non-normality of errors. Analyses were performed with linear mixed-effect models in order to account for the nested design of our experiment (ambient and reduced earthworm density subplots nested within plots). For this we used the *lme* function implemented in the *nmle* package [51]. Analyses of variation in the infiltration capacity were performed for each month separately. Starting from a constant null model with plot identity as a random factor, we added the design variables of the Jena Experiment: block (as a factor, BL; 1, 2, 3, 4), plant species richness (log-linear term; SR; 1, 4, 16), plant functional group richness (linear-term; FG; 1, 2, 3, 4) and the earthworm treatment (E) as well as the interaction terms E×SR and E×FG as fixed effects and assessed their significance using likelihood ratio tests (L-ratio). 95% confidence intervals for fixed effects were calculated based on the full model. Contrasts for the presence/absence of grasses (GR; 0, 1), legumes (LEG; 0, 1), small herbs (SH; 0, 1) and tall herbs (TH; 0, 1) were fitted in series of alternative models after accounting for block, plant species and functional group richness effects. Confidence intervals for these contrasts are based on the separate models. As the effects of the presence (or absence) of certain plant functional groups on the response variable were considered as post-hoc tests, the respective p-values were adjusted according to the Holm procedure to avoid inflation of Type I error rates [52]. We used simple linear regressions to analyse the influence of texture (sand fraction in 0–10 cm depth), and soil water content before the measurement on the infiltration capacity.

Path analysis was used to investigate how the total earthworm biomass extracted from the earthworm reduction subplot in September, soil texture and the presence/absence of legumes and grasses directly and indirectly affected the infiltration capacity on subplots with reduced (−ew) and ambient (+ew) earthworm densities in October. The impact on infiltration capacity in reduced and ambient earthworm density subplots was calculated in a separate analysis. Path analysis allows testing direct and indirect relationships between variables in a multivariate approach [53]. Hence, by using path analysis we were able to test if certain plant functional groups, such as legumes and grasses, directly influence infiltration capacity or if infiltration is indirectly influenced by other variables such as changes in earthworm biomass. In the path analysis, arrows represent causal relationships, while rectangles represent manipulated (grasses and legumes) or measured variables (sand content, earthworm biomass and infiltration capacity). Non-signifcant Chi^2^-test (p>0.05), low AIC and low RMSEA indicating an adequate model fit [53]. Beginning with the full model (including all possible pathways) the models were improved by stepwise removing of unimportant relationships based on AIC values [54]. Standardized path coefficients were derived based on the correlation matrix of standardized variables. Path analysis was performed using AMOS 5 (http://amosdeveleopment.com).

## Results

### Effect of Soil Texture and Moisture on Infiltration

**Figure 1:**
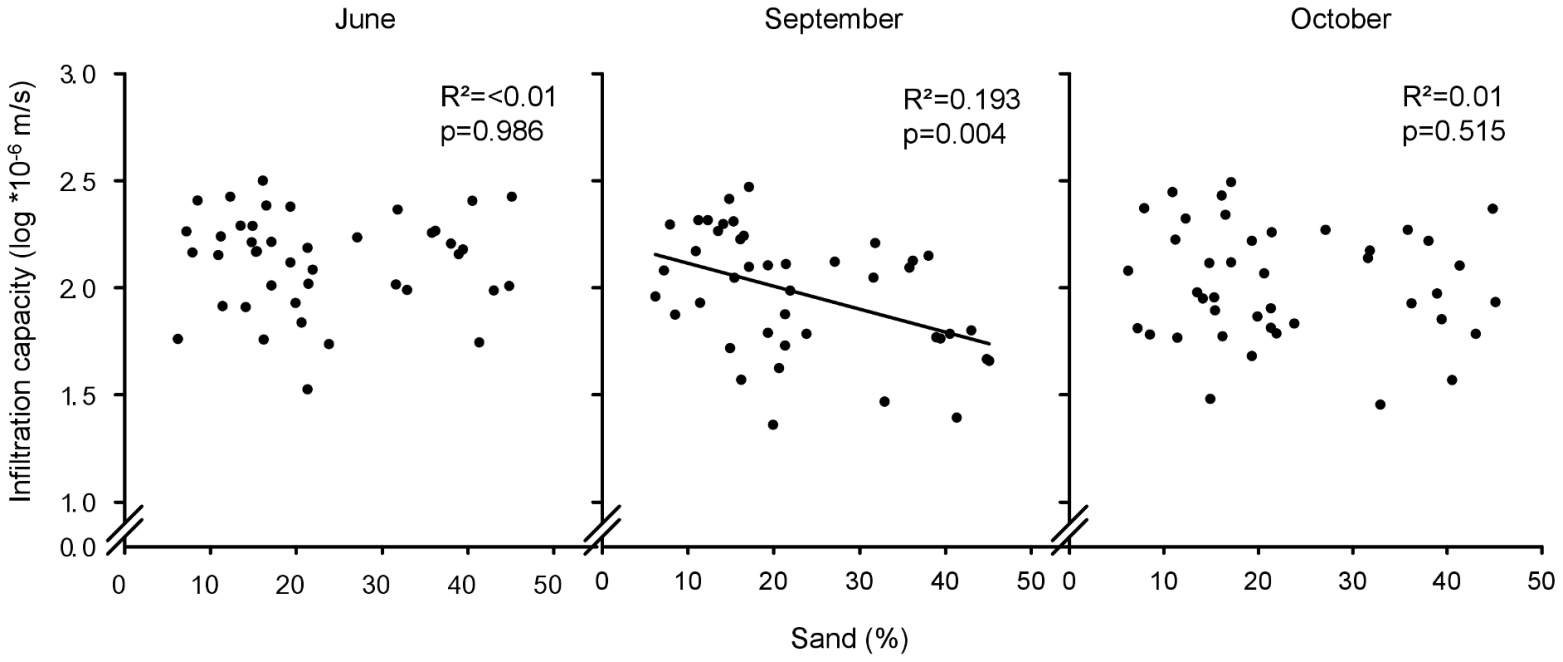
Relationship between infiltration capacity and soil texture for June, September, and October. Plot-level infiltration capacity at saturation plotted against soil texture (sand fraction in 0–10 cm depth in %) for the month June, September and October (as indicated). The regression line indicates significant relationships.

The infiltration capacity in June and October did not differ between the four blocks (Table 2) encompassing the texture gradient. In September, infiltration capacity increased systematically for *ψ*_M_ = 0 and −0.02 m from block 1 (71.34±11.50 and 49.23±8.55 *10^−6^ m/s) to block 4 (143.65±21.73 and 100.10±14.50 *10^−6^ m/s). When replacing the variable ‘block’ with ‘sand fraction in 0–10 cm depth’ as a covariate in our analysis, texture became significant in September (95% CI = [−1.68 to −0.34], Table S2), but not in June and October. Surprisingly, the infiltration capacity at saturated conditions decreased with increasing sand content (Figure 1, centre). However, when testing only plots without legumes, we did not find a significant correlation between sand content and infiltration capacity (data not shown). Additionally, there was a significant interaction between sand content and legumes (95% CI = [−2.92 to −0.56], Table S2), highlighting the differential effect of legumes with varying soil texture. In our experiment the initial soil moisture measurements did not correlate significantly with the infiltration capacity (log *10^−6 ^m/s) at matric potential zero in June (r = −0.207, p = 0.806), September (r = 0.439, p = 0.078) and October (r = 0.003, p = 0.993). In order to assure that the latter result was not artefacts stemming from the non-orthogonal design of the observed plots, we also tested for confounding correlations between the texture (sand content) and the presence and absence of legumes. There was no correlation (r = −0.117, p = 0.472).

**Table 2:** Results of linear mixed-effects models of the infiltration capacity for June, September and October.

	June					September				October					
Source	df	0 m		−0.02 m		0 m		−0.02 m		0 m		−0.02 m			
		L-ratio	p	L-ratio	p	L-ratio	p	L-ratio	p	L-ratio	p		L-ratio	p	
BL	3	4.05	0.256	4.02	0.259	**10.77**	**0.013**	**11.76**	**0.008**	2.89	0.409		2.92	0.403	
SR (log-linear)	1	0.28	0.596	0.12	0.724	0.04	0.834	0.06	0.800	0.76	0.385		0.50	0.480	
FG	1	0.70	0.403	2.43	0.119	0.03	0.858	<0.01	0.991	0.05	0.825		0.03	0.870	
GR	1	0.31	0.994	0.65	1.00	**4.27**	**0.116**↓	**4.62**	**0.096**↓	**6.39**	**0.045**↓		**7.59**	**0.018**↓	
LEG	1	0.72	0.639	0.76	1.00	**7.60**	**0.023**↑	**8.96**	**0.012**↑	**10.95**	**<0.001**↑		**10.53**	**0.004**↑	
SH	1	1.98	0.934	0.70	1.00	1.46	0.454	1.36	0.488	2.01	0.314		1.70	0.386	
TH	1	1.02	0.934	0.72	1.00	0.24	0.627	0.10	0.758	0.33	0.563		0.58	0.446	
E	1	**4.14**	**0.042**↑	1.89	0.169	0.23	0.630	0.46	0.498	0.88	0.349		0.34	0.557	
E×SR(log-linear)	1	1.33	0.248	0.19	0.664	0.21	0.645	0.14	0.706	**4.69**	**0.030**		**5.50**	**0.019**	
E×FG	1	0.60	0.437	1.80	0.406	0.26	0.614	0.49	0.484	1.24	0.264		**12.95**	**0.002**	

Infiltration capacity as affected by block (BL), plant species richness (SR), plant functional group richness (FG), grasses (GR), legumes (LEG), small herbs (SH), tall herbs (TH), and earthworm treatment (E) in June, September and October separately for the matric potentials *ψ*_M_ = 0 m and *ψ*_M_ = −0.02 m.

Models were fitted by stepwise inclusion of fixed effects. Likelihood ratio tests were applied to assess model improvement (L-ratio) and the statistical significance of the explanatory terms (p values). Models always included 84 observations (n) on 42 plots, df = degrees of freedom (df) for each of the predictor variables. For GR, LEG, SH and TH the adjusted p-values according to the Holm procedure are given in the table. Significant effects are marked in bold. Arrows indicate increase (↑) or decrease (↓).

### Effect of Plant Species Richness on Infiltration

**Figure 2:**
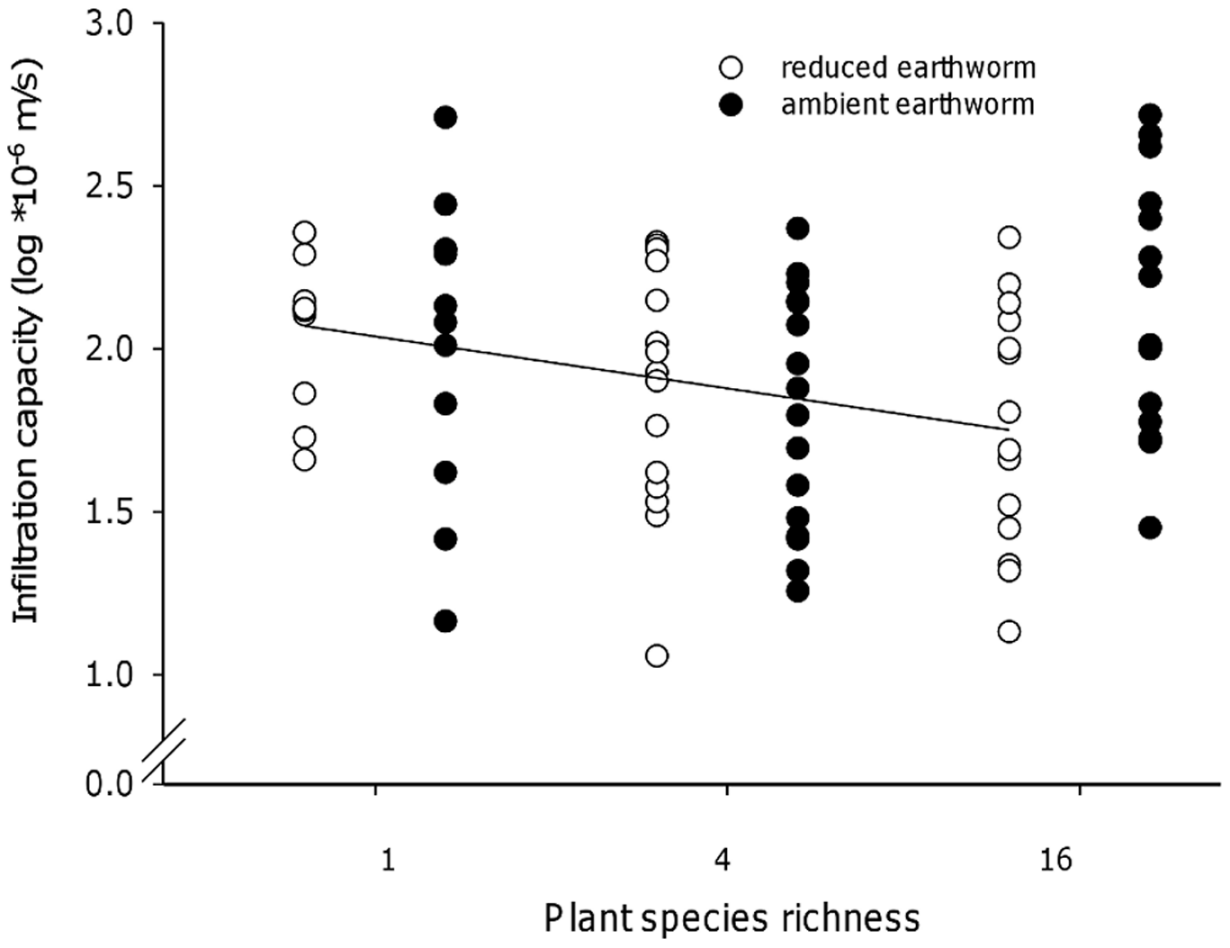
Interacting effects of plant diversity and earthworms on the infiltration capacity in October. Effect of plant species richness (1, 4, 16 species) and earthworm treatment (ambient earthworm density subplots and reduced earthworm density subplots) on the infiltration capacity at saturation. The regression line indicates a negative correlation between plant species richness and infiltration capacity on subplots with reduced earthworm densities (r = 0.33, p = 0.036).

Plant species richness (1, 4 and 16 species) did not significantly affect the infiltration capacity at any sampling date (Table 2). However, we observed a significant interaction effect between earthworms and plant species richness in October (95% CI = [0.072 to 0.280], Table 2), which was mainly caused by the mixture of 16 species (Figure 2). Additionally, the infiltration capacity was negatively correlated with plant species richness on plots with reduced earthworm densities (r = 0.332, p = 0.036). For infiltration on ambient earthworm density subplots a weak positive trend with plant species richness (r = 0.217, p = 0.166) was observed.

### Effects of Plant Functional Groups on Infiltration

**Figure 3:**
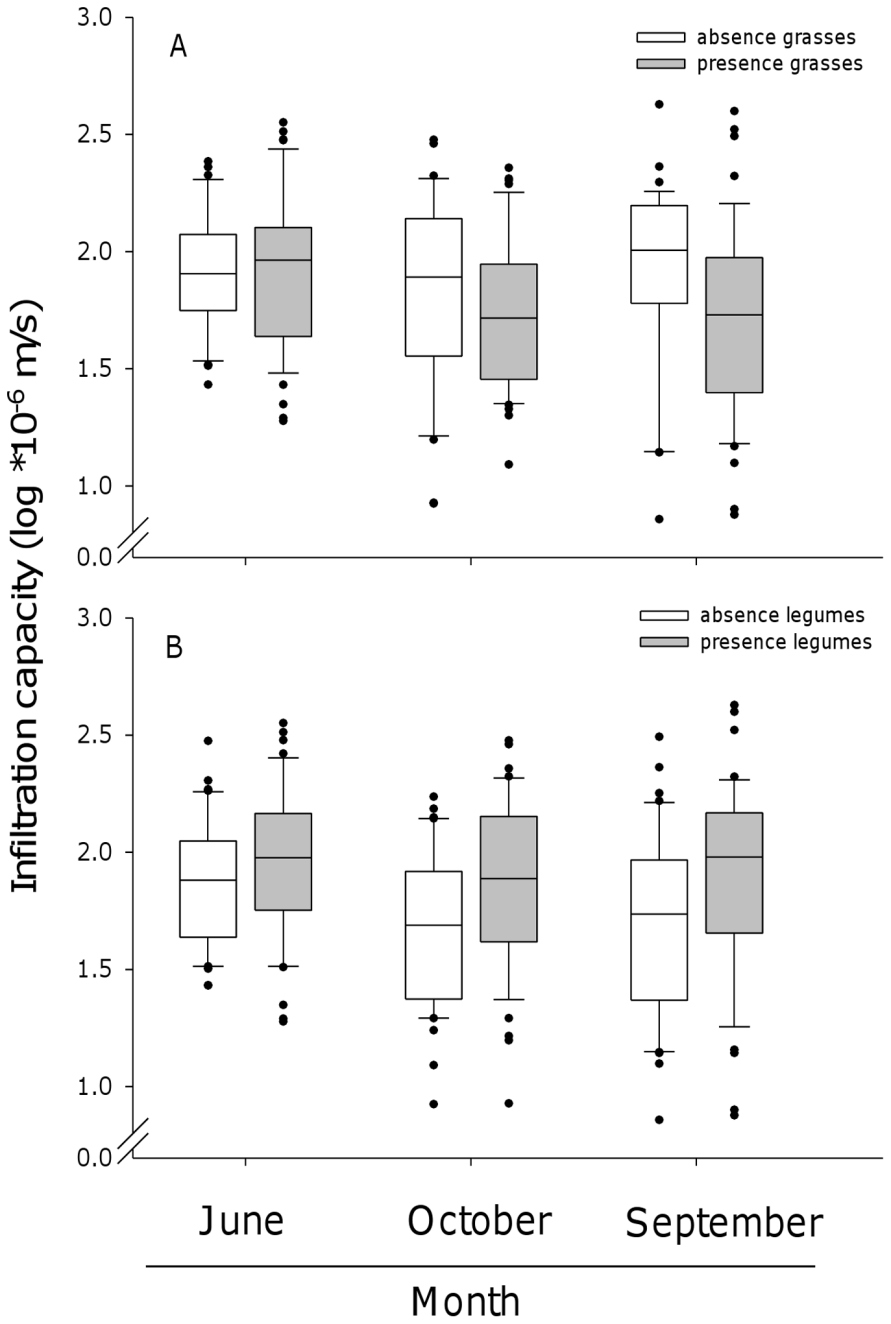
Effects of certain plant functional groups (presences of legumes and grasses) on the infiltration capacity. Variations in infiltration rates in June, September, and October as affected by (A) the presence of grasses, and (B) the presence of legumes at saturation. The box plots represent the median, the ends of the boxes defined the 25 and 75 quartiles. The error bars show the 10th and 90th percentiles of the data. Black dots outside of the 10th and 90th percentiles represent outliers.

Infiltration capacity was significantly affected by the presence of certain plant functional groups, such as grasses and legumes when fitted after BL and SR, however functional group richness had no effect on infiltration capacity. In September, infiltration capacity increased at *ψ*_M_ = 0 m by 39% (95% CI = [0.07 to 0.423]) and *ψ*_M_ = −0.02 m by 40% (95% CI = [0.14 to 0.53]) in the presence of legumes (adjusted p-value = 0.023 and 0.012, respectively), while the presence of grasses caused only a small, non-significant decrease in infiltration capacity (adjusted p-value = 0.116 and 0.096, respectively; Figure 3, Tables 2 and S3). In October, both legumes and grasses significantly affected the infiltration capacity: in presence of legumes infiltration capacity increased at *ψ*_M_ = 0 m by 36% (95% CI = [0.14 to 0.53]) and *ψ*_M_ = −0.02 m by 38% (95% CI = [0.13 to 0.56]), while it decreased in the presence of grasses at *ψ*_M_ = 0 m by 23% (95% CI = [−0.46 to −0.06]) (adjusted p-value = <0.001 and 0.004, respectively) and at *ψ*_M_ = −0.02 m by 27% (95% CI = [−0.41 to −0.09]) (adjusted p- value = 0.045 and 0.018, respectively; Figure 3, Tables 2 and S3). No significant effects of plant functional groups were detected in June (Tables 2 and S3).

Because we worked on a short diversity gradient, the design is not completely orthogonal with respect to plant species richness and plant functional group number (r = 0.439, p<0.001), However, plant species richness and functional groups did not explain a signficant proportion of variation in infiltration capacity (Table 2) and therefore this correlation did not affect our results. Furthermore, there exist a positive relationship between plant species richness and presence of grasses (r = 0.290, p = 0.062) and legumes (r = 0.223, p = 0.155) respectively. In order to test whether our results were confounded by this correlation, we alternatively fitted legumes and grasses prior and after plant species richness, and found that fitting order did not affect the results (analyses not shown).

### Effect of Earthworms on Infiltration

The biomass of endogeic earthworms accounted for 59% and 49% of total earthworm biomass in spring and fall, whereas the biomass of anecic species (*L. terrestris*) accounted for 36% and 49% of total biomass in spring and fall, respectively. The total number and biomass of earthworms increased significantly from spring (26.88±2.29 individuals m^−2^ and 13.86±2.03 g m^−2^, respectively) to fall (40.55±4.02 individuals m^−2^ and 25.78±2.35 g m^−2^, respectively; Table 3). Total earthworm biomass increased slightly, but not significantly in the presence of legumes in spring (t-test, p = 0.338), but significantly in fall (t-test, p = 0.001). In presence of grasses, total earthworm biomass decreased slightly but not significantly in spring and in fall (analyses not shown). For both extraction dates earthworm biomass did not correlate with plant species richness (analyses not shown).

**Table 3:** Number (individuals m^−2^) and biomass (g m^−2^) of earthworms of the Jena Experiment.

Earthworms	March	September
**Number of anecics**	3.14±0.53	5.47±0.63
**Biomass of anecics**	4.98±1.03	12.74±1.37
**Number of endogeics**	21.51±2.25	34.05±3.67
**Biomass of endogeics**	8.13±1.01	12.62±1.51
**Number total earthworms**	26.88±2.92	40.55±4.02
**Biomass total earthworms**	13.86±2.03	25.78±2.35

Total number and biomass of earthworms included also unidentifiable earthworms and the invasive earthworm *Lumbricus castaneus*.

Shown are the means (±1 SE) across plots in 2011 for the extraction dates in spring (March) and autumn (September).

**Figure 4:**
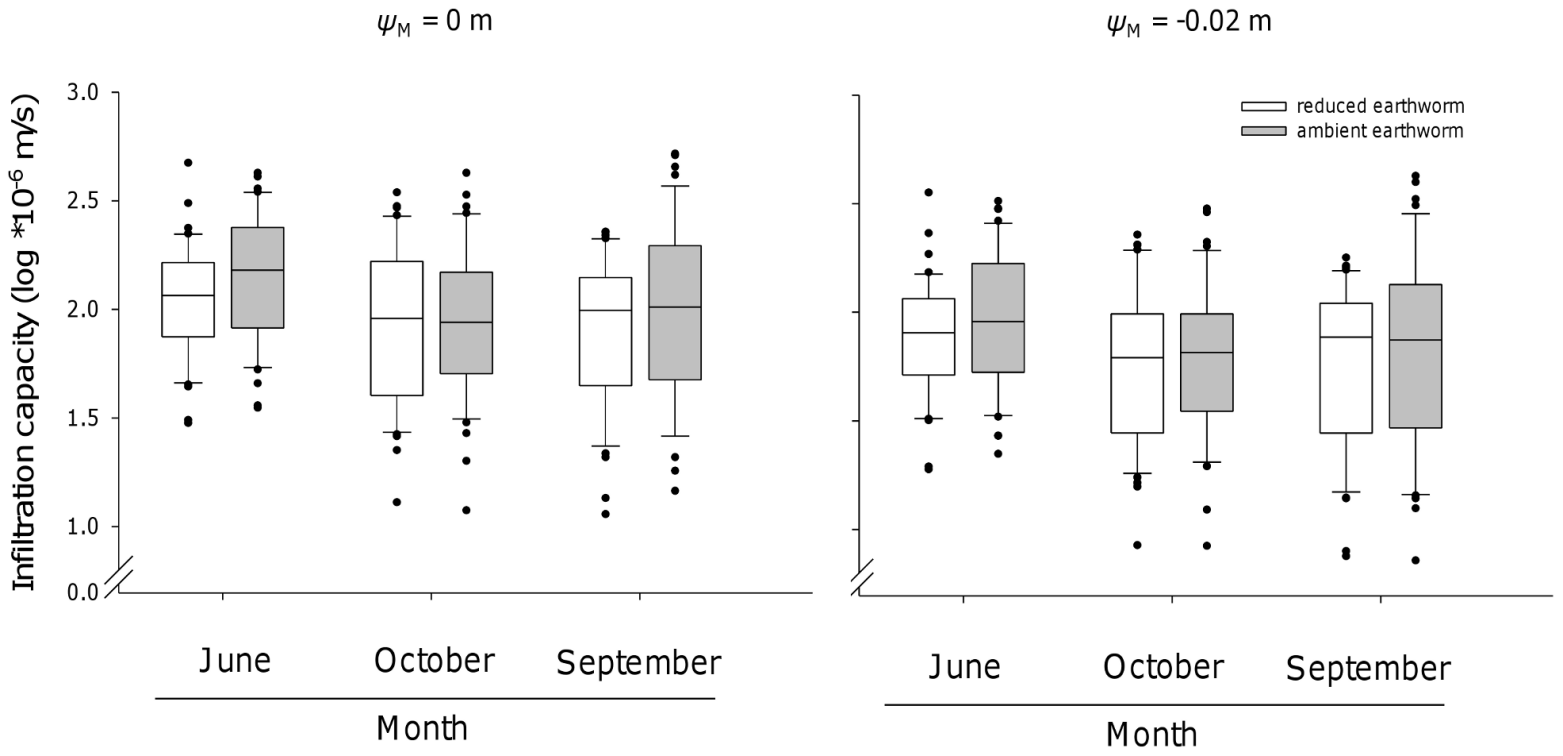
Effects of earthworms on the infiltration capacity. Variations in infiltration rates in June, September, and October as affected by earthworms (ambient and reduced earthworm density) for applied matric potential *ψ*_M_ = 0 m (left panel) and *ψ*_M_ = −0.02 m (right panel). The box plots represent the median, the ends of the boxes defined the 25 and 75 quartiles. The error bars show the 10th and 90th percentiles of the data. Black dots outside of the 10th and 90th percentiles represent outliers.

In June 2011, the infiltration capacity was only marginally increased (linear mixed-effect models: n = 84, L-ratio = 4.14, p = 0.042, 95% CI = [−0.14 to −0.31]) in ambient earthworm density subplots (171.24±16.20 *10^−6^ m/s) compared to the reduced earthworm density subplots (128.75±12.72 *10^−6^ m/s). With decreasing matric potential (*ψ*_M_ = −0.02 m, exclusion of pores >1.5 mm) the effect of earthworms disappeared (Figure 4, Table 2, S3). For all other sampling dates and matric potentials, the earthworm treatment did not significantly affect infiltration capacity (Figure 4, Tables 2 and S3), while the earthworm biomass affected the infiltration capacity (see path analysis).

### Path Analysis

**Figure 5:**
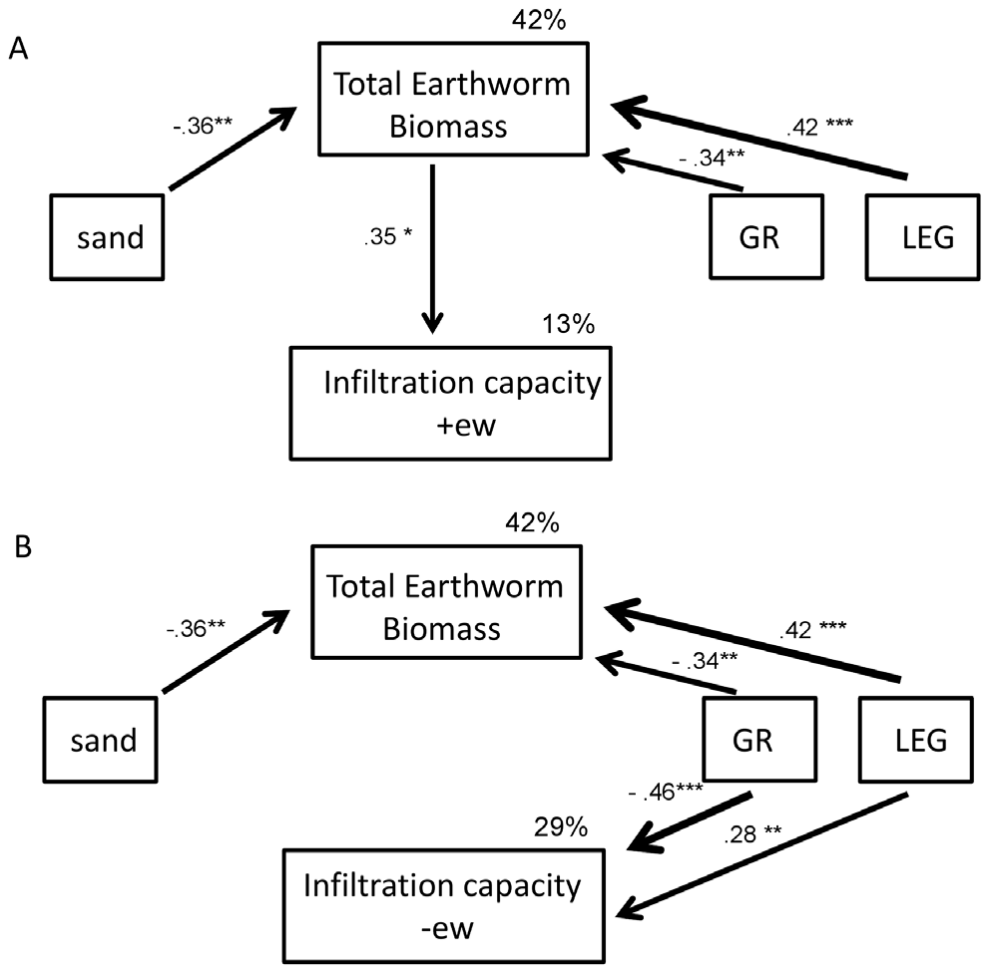
Path analysis of factors explaining infiltration capacity on plots with ambient or reduced earthworm density. Relationships between total biomass of earthworms (earthworm biomass), texture (sand in 10 cm depth) and plant functional groups (GR, grasses; LEG, legumes) for (A) infiltration rate on subplots with ambient (infiltration capacity +ew) and (B) reduced earthworm densities (infiltration capacity –ew) in October. The total earthworm biomass was extracted in September. Standardized path coefficients are given next to path arrows. Unexplained variation is denoted with e1–e3; *p≤0.05, **<0.01, ***p = 0.001. For details see text.

The path analysis supported the results of the linear mixed-effect model approaches showing strong grass and legumes effects on the infiltration capacity. In addition, we could identify possible mechanisms shaping the infiltration capacity in ambient and reduced earthworm density subplots. The initial model for October (AIC = 49.10, Figure S1) was improved as described in the method section. In October the final model explained 13% of the infiltration capacity on subplots with ambient earthworm density (Figure 5A; χ^2^ = 1.80, p = 0.937, AIC = 29.80) and 29% of the infiltration capacity on subplots with reduced earthworm density (Figure. 5B; χ^2^ = 1.10 p = 0.954, AIC = 31.10). The total earthworm biomass increased in the presence of legumes and decreased in the presence of grasses. Earthworm biomass decreased with increasing sand content. Increasing total earthworm biomass increased the infiltration capacity on ambient earthworm density subplots directly (Figure 5A). Grasses decreased and legumes increased infiltration capacity on subplots with reduced earthworm biomass (Figure 5B). In summary, grasses had a stronger direct effect on the infiltration capacity on subplots with reduced earthworm density, whereas legumes had a stronger indirect effect on infiltration capacity on subplots with ambient earthworm density by increasing earthworm biomass (Figure 5B). For the infiltration on ambient earthworm density subplots we additionally included different ecological earthworm groups (anecic and endogeic) instead of the total earthworm biomass in separate models to disentangle the effects of different ecological earthworms groups on the infiltration capacity and to test the different effects of sand content and the presence/absence of legumes and grasses on them. Including only the biomass of anecic earthworms instead of total earthworm biomass in additional models, the effect of grasses and sand content disappeared, but the effect of legumes on the biomass of anecic earthworms increased (Figure S2A). In contrast, including the biomass of endogeic species instead of the total biomass, we observed similar results as for the total earthworm biomass model (Figure S2B).

## Discussion

### Effect of Earthworms on Infiltration

Our results indicate that biotic factors play a decisive role for soil hydraulic soil properties near saturation. In June infiltration capacity slightly increased on subplots with ambient earthworm densities compared to subplots with reduced earthworm densities. Remarkably, this effect was mainly caused by plots in block 1 and block 4 (data not shown) suggesting local changes of earthworm activity and considerable spatial variation due to texture and plant type that interact with infiltration [55]. In general, burrows of anecic species such as *L. terrestris* but also those of adult endogeic species such as *A. caliginosa* are larger than 2 mm in diameter and this may explain why only water flow through larger pores (>1.5 mm, at matric potential *ψ*_M_ = 0 m close to saturation), but not through smaller pores was directly affected by the presence of earthworms. This is also in line with the non-capillary nature of earthworm casts. Earthworm presence, particularly that of anecic species forming vertical burrows, facilitates larger pores conducting water (macropore flow) and contributes to infiltration when water is supplied in large quantities (tension free conditions) [56].

We measured infiltration capacity at elevated soil saturation, and it could be argued that saturation will never be reached in most natural soil environments. However, irrigation experiments with dye indicate that macropores, such as those formed by anecic earthworms, are activated at realistic rain intensities [19], [57], [58]. Beven and Germann [15] reported that rainfall intensities of 1–10 mm h^−1^ can initiate macropore flow. Applying an arbitrary threshold of 5 mm h^−1^ [59] to the high resolution (10 min) rainfall record of our field site (2003 to 2011) indicates that about 30% of the total rainfall could deliver macropore flow. One of the pressing questions for future work will be on how this macropore flow potential relates to actual water flow at greater depth. Furthermore, it is expected that precipitation patterns change due to climate change with decreasing precipitation in summer and increasing precipitation in fall/winter. It is therefore likely that higher frequency of extreme precipitation events increases the proportion of heavy rainfalls [60]. Macropores formed by earthworms and roots, and their interactions, thus likely to become more important for buffering strong precipitation events in the future.

### Effects of Plant Functional Groups on Infiltration

At the end of the growing season infiltration capacity was strongly affected by the presence of certain plant functional groups, such as grasses and legumes. Legumes increased and grasses decreased infiltration capacity (Figure 3, Table 2). This is consistent with results of Archer et al. [61], who reported that legumes increased and grasses decreased hydraulic conductivity. Further, the significant effect of legumes was more pronounced at *ψ*_M_ = 0 m, whereas the effect of grasses was more pronounced effect at smaller matric potential (*ψ*_M_ = 0.02 m). It has been shown that decaying tap-roots of legumes form stable macropores and hence increase infiltration [27], [62]. This observation was supported by Mytton et al. [63], who showed that water infiltration was higher under clover compared to grass due to a higher fraction of soil pores greater than 60 µm with porosity being equal. Additionally, several studies showed that water flow through soil is enhanced by legumes or legumes-grass mixtures compared to pure grass stands due to root proliferation, which increased soil organic matter content and favored soil fauna such as earthworms [64]. The fibrous and rhizomatous roots of grass species tend to reduce infiltration by clogging soil pore space and blocking water flow [61].

### Path Analysis

Path analysis helped to address underlying mechanism: the effect of legumes on infiltration capacity in ambient (control) earthworm density plots may be indirect by enhancing earthworm biomass (Figure 5A), suggesting that earthworm performance benefits from the presence of legumes [34], [35]. Additionally, the path analysis indicated that grasses directly influenced the infiltration capacity on subplots with reduced earthworm densities, while indirect effects of grasses via decreasing earthworm biomass were less important (Figure 5). For the Jena Experiment it was shown by Pérès et al. [29] that root biomass is strongly increased in presence of grasses while, as mentioned above, legumes signifcantly increased earthworm biomass (Figure 5A). This suggests that the observed enhanced infiltration in plots with legumes was probably associated with a larger number of macropores caused by earthworms [65]. These results are in agreement with the findings of Abbott and Parker [66], who reported an increased infiltration of water due the activity of the geophagous earthworm species *Microscolex dubuis* in the presence of clover mulch. By contrast, decreased infiltration in grass plots was probably due to fine roots clogging soil pores. Thus, our results suggest that the presence of grasses and legumes affected hydraulic conductivity via different mechanisms, directly presumably via root activity and indirectly via altering earthworm biomass. The observed effects increased during the vegetation period (Table 2), probably due to the progressive increase of earthworm (Table 3) and root biomass [67].

The different ecological earthworm groups differ in forming of macro- and microaggregates by changing soil structure features (aggregate size, stability and soil organic content) and porosity (pore size distribution) [28], [68]. Interestingly, in October anecic earthworms were positively affected by the presence of legumes, whereas endogeic earthworms were negatively affected by sand content and by the presence of grasses. Additionally, both endogeic and anecic earthworm had a significant effect on the infiltration capacity, with a more pronounced effect of anecic earthworms (Figure S2). This is in line with other studies, which showed that the deep dwelling anecic earthworm enhance water infiltration rates [24], [69], while horizontal pores formed by endogeic earthworms limits the effectiveness in water flow through soil [70]. Generally, endogeic earthworms are considered as the major group improving soil aggregation, while anecic or compacting species destabilizes the soil due to their casting activity [28]. As shown by Lee and Foster [71] a mix of endogeic and anecic earthworms supports soil structural health.

In part our results are in contrast to other studies [72]–[74], which found a direct impact of earthworm treatment on infiltration. Studies on the influence of earthworms on infiltration capacity usually ignore gradients of texture and plant functional groups. This gradient increased the variance in our data and affected the earthworm populations in the subplots. However, our observation area was also rather small (240 cm^2^) and the setup prevented from conducting additional measurements. Also, we compared subplots with ambient to subplots with reduced earthworm densities contrasting earlier studies which compared ambient conditions (by earthworm addition) with a control (no earthworms) [75], [76]. While our setup has the advantage to better reflect ‘natural’ conditions, it may have caused a comparatively smaller contrast between treatments. Since the used octet extraction method cannot remove earthworms completely (extraction reduced the surface activity of earthworms by about 38% five weeks after the last manipulation [77]), the differences between the treatments might not have been as strong as in other experiments, and re-colonization of earthworm reduction subplots may have weakened the contrast further. Unfortunately, we cannot exclude completely that extraction efficiency varies depending on plant community properties, because there exist no detailed studies that evaluated the extraction efficiency of the octet method as a function of plant community structure. Effects of earthworms on plant performance and the resulting impact on soil hydraulic properties depend also on other soil factors, such as texture and bulk density, leading to substantial spatial variation and interaction which probably masked earthworm effects. More observations on the pore structure in the different treatments are necessary to validate the proposed complex interactions in soil processes leading to the observed infiltration patterns.

### Effect of Plant Species Richness on Infiltration

In contrast to the pronounced effects of certain plant functional groups, such as legumes and grasses, plant diversity measures (plant species and functional group richness) had only small effects on the infiltration capacity and earthworm performance. In October, we found that plant diversity had only a marginally significant effect on the infiltration capacity on subplots with ambient earthworm density, but plant species diversity affected infiltration capacity significantly on subplots with reduced earthworm density, presumably due to plant roots clogging macropores [78], [79]. This is supported by data on the relationship between plant diversity and standing root mass in the Jena Experiment, showing that root biomass also increased with diversity level [38], [80]. Additionally, infiltration capacity decreased over the growing season (data not shown) presumably by the growing roots clogging macropores [79]. Angulo-Jaramillo et al. [81] observed a decrease of hydraulic conductivity caused by sealing of interconnected pores at the soil surface. However, on subplots with ambient earthworm densities this clogging may have been counteracted by the activity of earthworms. These results suggest that water flow through soil is more strongly affected by earthworm biomass which is regulated by the presence of certain functional groups (particularly by legumes) then by loss of single plant species along the observed gradient.

### Effect of Soil Texture and Moisture on Infiltration

Grain size distribution strongly influences hydraulic properties of porous media, and therefore texture has often been related to hydraulic conductivity [9], [82]. However, in our experiment saturated and near-saturated infiltration capacity in June and October were not affected by soil texture (Figure 1). Surprisingly, in September, infiltration capacity varied with soil texture, but unexpectedly it was lowest in coarse textured soils. However, the results are in agreement with Jarvis and Messing [12] and Lin et al. [3], who found higher hydraulic conductivity in finer as compared to coarser textured soils due to well-developed soil structure (earthworm burrows, root channels) and a high degree of macroporosity. As detailed above, legumes increased infiltration capacity, and interestingly the negative correlation between sand content and infiltration capacity disappeared when removing plots containing legumes. This is probably a result of the positive relationship between legumes and earthworms and resulting effects on infiltration. An alternative explanation for increased infiltration in the presence of legumes could be that roots affected the formation of aggregates and structure, particularly so in finer textured soils [5], [28]. Kördel et al. [83] suggested that the formed macropores are less stable over time in sandy soil. However, investigations on soil stability in the Jena Experiment itself [29] suggest the opposite effect, with legumes decreasing soil aggregate stability (indirectly, by increasing earthworm biomass and decreasing plant root biomass). At the same time, we found a lower number of earthworms in more sandy soils in 2011 (data not shown). This is in line with previous studies [84], [85] showing that sandy soils support smaller earthworm populations than clayey soils resulting in lower hydraulic conductivity in sandy soils. Thus, our results may be explained by the promotion of earthworm abundance by legumes and finer soil texture, but further research is necessary to support these findings. Our results underline the importance of soil structure as influenced by biotic processes and corroborate the findings of Bonsu [86] who suggested that the texture based calculation underestimates the hydraulic conductivity, in particular in fine textured soils.

One important source of error that can dominate or suppress other factors is the initial soil moisture content. Several studies have shown that the infiltration through soil is correlated with the initial soil moisture content [87], [88]. This relation was not expected in our setup, since the soil moisture is controlled by fixing the infiltration pressure head. At the beginning of an infiltration experiment, low initial soil moisture content enhanced the water flow through the soil because of larger gradients in matric potential and filling up of the soil storage. The influence of initial moisture content decreased over the duration of the experiment, when the pores probably were filled and infiltration reached a steady state [89]. A potential error source is finishing the experiment before steady infiltration rates are reached in dry soils and hence overestimating infiltration capacity. Thus, we checked whether infiltration rates were biased by initial soil moisture and this was not the case. Thus our infiltration measurements were performed correctly. Another factor potentially affecting infiltration rates is soil hydrophobicity. Hydrophobic exudates are produced by plant roots and soil microbes. Soto et al. [90] observed that the soil showed a tendency to be water repellent if the volumetric water content fell below *θ*_c_ = 22% for medium textured soils, the so called ‘critical soil water content’ [91]. The majority of the measured volumetric water contents exceeded this threshold (data not shown). Since most of our observed effects enhanced during the growing season, while the chance for hydrophobicity decreased, we conclude that water repellency did not affect our results.

### Conclusions

Despite large spatial variability of soil hydraulic properties, biotic factors emerged as significant agents for infiltration. The presence of legumes increased and the presence of grasses decreased infiltration capacity over the course of the growing season. The path analysis suggests that modifications in hydraulic conductivity are probably due to (i) roots directly, modifying the pore spectrum and (ii) indirectly, suppressing (grasses) or enhancing (legumes) earthworm activity. The results suggest that earthworm biomass is synchronized with other plant related processes such as root growth. Thus the observed effects of plant functional groups are attributable to earthworm and root activity.

Most predictions of near surface soil hydraulic properties are based on easily accessible soil properties such as soil texture. Our results suggest that biotic effects, especially the presence of certain plant functional groups affecting earthworm biomass, shape hydraulic conductivity and may even reverse effects of texture. Therefore, for explaining variations in hydrological processes, such as infiltration capacity, the structure of soil fauna and plant communities need to be considered.

## Supporting Information

Figure S1 Initial path analysis of factors explaining infiltration capacity on subplots with ambient or reduced earthworm density. Relationships between earthworm biomass (total, anecic or endogeic as indicated), texture (sand in 10 cm depth) and functional groups (GR, grasses; LEG, legumes) and infiltration capacity at saturation for ambient (+ew) or reduced (−ew) earthworm density plots (as indicated).
(TIF)

Figure S2 Path analysis of the effects of the ecological earthworm groups (anecic and endogeic) on infiltration capacity. Path analysis showing the relationships between (A) endogeic and (B) anecic earthworm with texture (sand in 10 cm depth) and plant functional groups (GR, grasses; LEG, legumes) for infiltration capacity on subplots with ambient (+ew) earthworm densities in October. Standardized path coefficients are given next to path arrows. Unexplained variation is denoted with e1–e3; *p≤0.05, **<0.01, ***p = 0.001. For details see text.
(TIF)

Table S1 Block-wise variations in soil parameters (clay, silt and sand content) of the upper 10 cm. Plots were assembled into four blocks with block 1 nearest the river Saale and block 4 furthest from the river.
(DOCX)

Table S2 Summary of mixed effects models for infiltration capacity at saturation in September. Results for the infiltration rate as affected by sand content in 0–10 cm depth (Sand), plant species richness (SR), plant functional group richness (FG), grasses (GR), legumes (LEG), small herbs (SH), tall herbs (TH), interaction Sand×LEG and earthworm treatment (E) as well as the interaction terms E×SR and E×FGfor measurements in September.
(DOCX)

Table S3 Summary of the analysis of infiltration capacity at *ψ*_M_ = 0 m and 0.02 m. Estimates (est.) with 95% confidence intervals (lower and upper) for the effects of block (Block), plant species richness (SR), plant functional group richness (FG), grasses (GR), legumes (LEG), small herbs (SH), tall herbs (TH), and earthworm treatment (E) in June, September and October for the matric potentials *ψ*_M_ = 0 m and 0.02 m.
(DOCX)
