# Supplementary figures and images for: How Do Earthworms, Soil Texture and Plant Composition Affect Infiltration along an Experimental Plant Diversity Gradient in Grassland?
Source: PLoS One. 2014 Jun 11;9(6):e98987. doi: 10.1371/journal.pone.0098987 (PMC4053431; doi:10.1371/journal.pone.0098987)

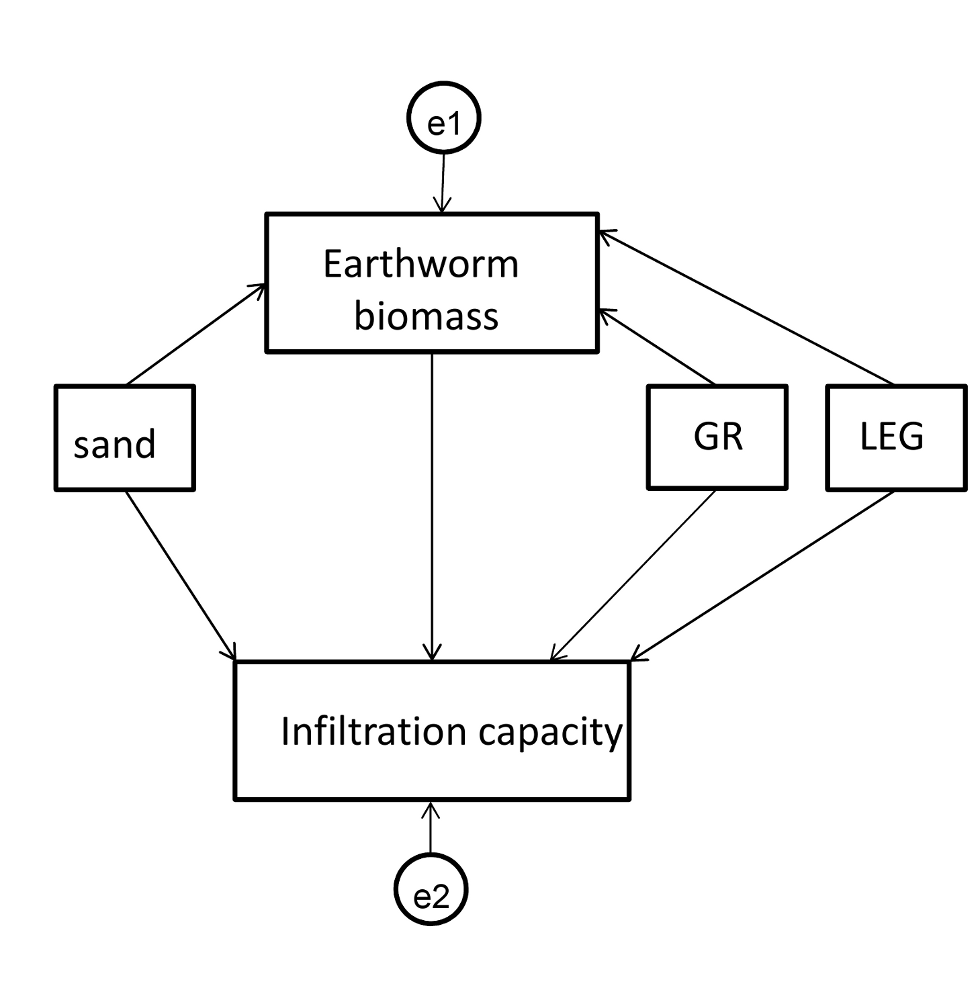

Supplement: Figure S1 — Initial path analysis of factors explaining infiltration capacity on subplots with ambient or reduced earthworm density. Relationships between earthworm biomass (total, anecic or endogeic as indicated), texture (sand in 10 cm depth) and functional groups (GR, grasses; LEG, legumes) and infiltration capacity at saturation for ambient (+ew) or reduced (−ew) earthworm density plots (as indicated). (TIF) [file pone.0098987.s001.tif]

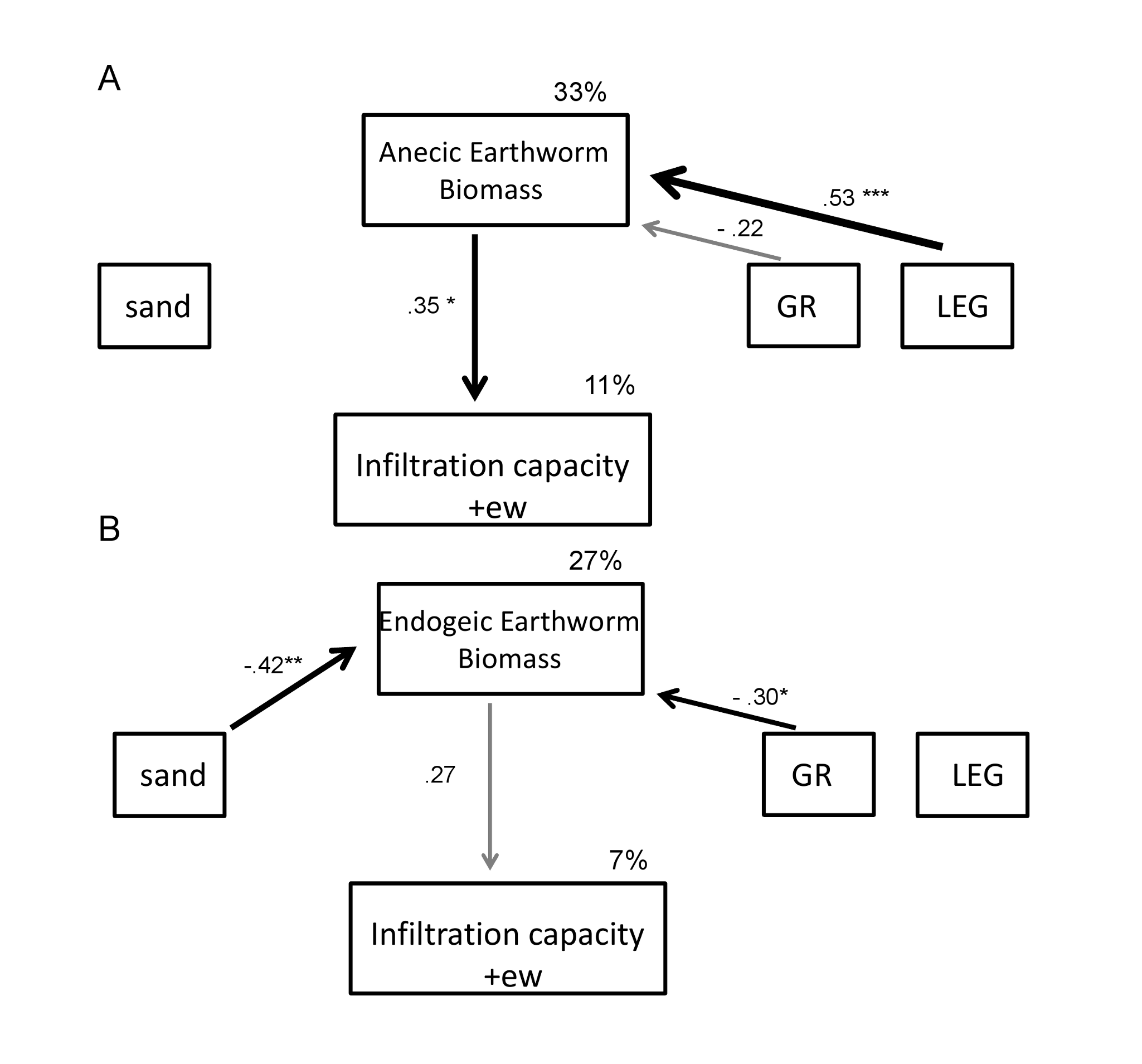

Supplement: Figure S2 — Path analysis of the effects of the ecological earthworm groups (anecic and endogeic) on infiltration capacity. Path analysis showing the relationships between (A) endogeic and (B) anecic earthworm with texture (sand in 10 cm depth) and plant functional groups (GR, grasses; LEG, legumes) for infiltration capacity on subplots with ambient (+ew) earthworm densities in October. Standardized path coefficients are given next to path arrows. Unexplained variation is denoted with e1–e3; *p≤0.05, **<0.01, ***p = 0.001. For details see text. (TIF) [file pone.0098987.s002.tif]
