# Supplementary material for: How Do Earthworms, Soil Texture and Plant Composition Affect Infiltration along an Experimental Plant Diversity Gradient in Grassland?
Source: PLoS One. 2014 Jun 11;9(6):e98987. doi: 10.1371/journal.pone.0098987 (PMC4053431; doi:10.1371/journal.pone.0098987)
Supplement: Table S1 — Block-wise variations in soil parameters (clay, silt and sand content) of the upper 10 cm. Plots were assembled into four blocks with block 1 nearest the river Saale and block 4 furthest from the river. (DOCX) [file pone.0098987.s003.docx]

**Table S1**. **Block-wise variations in soil parameters (clay, silt and sand content) of the upper 10 cm.** Plots were assembled into four blocks with block 1 nearest the river Saale and block 4 furthest from the river.

| **Soil abiotic parameters** | Block 1 | Block 2 | Block 3 | Block 4 |
| --- | --- | --- | --- | --- |
| clay | 16% | 21% | 22% | 23% |
| sand | 40% | 22% | 17% | 11% |
| silt | 44% | 57% | 61% | 66% |
