# Supplementary material for: How Do Earthworms, Soil Texture and Plant Composition Affect Infiltration along an Experimental Plant Diversity Gradient in Grassland?
Source: PLoS One. 2014 Jun 11;9(6):e98987. doi: 10.1371/journal.pone.0098987 (PMC4053431; doi:10.1371/journal.pone.0098987)
Supplement: Table S2 — Summary of mixed effects models for infiltration capacity at saturation in September. Results for the infiltration rate as affected by sand content in 0–10 cm depth (Sand), plant species richness (SR), plant functional group richness (FG), grasses (GR), legumes (LEG), small herbs (SH), tall herbs (TH), interaction Sand×LEG and earthworm treatment (E) as well as the interaction terms E×SR and E×FGfor measurements in September. (DOCX) [file pone.0098987.s004.docx]

**Table S2.** **Summary of mixed effects models for infiltration capacity at saturation in September.** Results for the infiltration rate as affected by sand content in 0-10 cm depth (Sand), plant species richness (SR), plant functional group richness (FG), grasses (GR), legumes (LEG), small herbs (SH), tall herbs (TH), interaction Sand x LEG and earthworm treatment (E) as well as the interaction terms E x SR and E x FGfor measurements in September.

|  | **September** | | |
| --- | --- | --- | --- |
| **Source** | 0 m | | |
|  | L-ratio | p |  |
| Sand | **8.41** | **0.004** | ↓ |
| SR (log-linear) | <0.01 | 0.969 |  |
| FG (linear) | 0.10 | 0.750 |  |
| GR | 2.73 | 0.294 |  |
| LEG | **6.96** | **0.033** | ↑ |
| SH | 1.34 | 0.494 |  |
| TH | 0.06 | 0.802 |  |
| Sand×LEG | **12.67** | **0.002** |  |
| E | 0.24 | 0.628 |  |
| E×SR(log-linear) | 0.21 | 0.646 |  |
| E×FG (linear) | 0.25 | 0.615 |  |

_Models were fitted by stepwise inclusion of fixed effects. Likelihood ratio tests were applied to assess model improvement (L ratio) and the statistical significance of the explanatory terms (p values). For GR, LEG, SH and TH the adjusted p-values according to the Holm procedure are given in the table. Significant effects are marked in bold. Arrows indicate increase (↑) or decrease (↓)._
