# Supplementary material for: How Do Earthworms, Soil Texture and Plant Composition Affect Infiltration along an Experimental Plant Diversity Gradient in Grassland?
Source: PLoS One. 2014 Jun 11;9(6):e98987. doi: 10.1371/journal.pone.0098987 (PMC4053431; doi:10.1371/journal.pone.0098987)
Supplement: Table S3 — Summary of the analysis of infiltration capacity at ψ M = 0 m and 0.02 m. Estimates (est.) with 95% confidence intervals (lower and upper) for the effects of block (Block), plant species richness (SR), plant functional group richness (FG), grasses (GR), legumes (LEG), small herbs (SH), tall herbs (TH), and earthworm treatment (E) in June, September and October for the matric potentials ψ M = 0 m and 0.02 m. (DOCX) [file pone.0098987.s005.docx]

**Table S3.** **Summary of the analysis of infiltration capacity at *ψ*_M_ = 0 m and 0.02 m**. Estimates (est.) with 95% confidence intervals (lower and upper) for the effects of block (Block), plant species richness (SR), plant functional group richness (FG), grasses (GR), legumes (LEG), small herbs (SH), tall herbs (TH), and earthworm treatment (E) in June, September and October for the matric potentials *ψ*_M_ = 0 m and 0.02 m.

|  | **June** | | | | | | |  | **September** | | | | | | |  | **October** | | | | | | |
| --- | --- | --- | --- | --- | --- | --- | --- | --- | --- | --- | --- | --- | --- | --- | --- | --- | --- | --- | --- | --- | --- | --- | --- |
| **Source** | 0 m | | |  | 0.02 m | | |  | 0 m | | |  | 0.02 m | | |  | 0 m | | |  | 0.02m | | |
|  | lower | est. | upper |  | lower | est. | upper |  | lower | est. | upper |  | lower | est. | upper |  | lower | est. | upper |  | lower | est. | upper |
| Intercept | 1.813 | 2.016 | 1.465 |  | 1.628 | 1.844 | 2.061 |  | 1.465 | 1.709 | 1.954 |  | 1.268 | 1.518 | 1.769 |  | 1.737 | 2.008 | 2.280 |  | 1.594 | 1.884 | 2.173 |
| Block2 | -0.236 | -0.056 | -0.082 |  | -0.238 | -0.050 | 0.136 |  | -0.082 | 0.130 | 0.344 |  | -0.066 | -0.151 | 0.368 |  | -0.092 | 0.148 | 0.374 |  | -0.093 | 0.148 | 0.374 |
| Block3 | -0.273 | -0.099 | 0.092 |  | -0.292 | -0.110 | 0.071 |  | 0.092 | 0.299 | 0.507 |  | 0.107 | -0.319 | 0.529 |  | -0.113 | 0.131 | 0.336 |  | -0.103 | 0.131 | 0.336 |
| Block4 | -0.111 | 0.068 | 0.097 |  | -0.134 | 0.052 | 0.239 |  | 0.097 | 0.311 | 0.524 |  | 0.122 | 0.339 | 0.556 |  | -0.040 | 0.198 | 0.426 |  | -0.043 | 0.198 | 0.426 |
| SR (log-linear) | -0.134 | -0.042 | -0.132 |  | -0.132 | 0.033 | 0.066 |  | -0.132 | 0.021 | 0.090 |  | -0.132 | 0.018 | 0.096 |  | -0.216 | -0.094 | 0.064 |  | -0.228 | -0.094 | 0.064 |
| FG | -0.039 | 0.047 | -0.080 |  | -0.043 | 0.049 | 0.141 |  | -0.080 | 0.024 | 0.090 |  | -0.082 | 0.025 | 0.131 |  | -0.153 | 0.044 | 0.088 |  | -0.169 | 0.044 | 0.088 |
| GR | -0.459 | 0.257 | -0.370 |  | -0.208 | 0.047 | 0.113 |  | -0.370 | -0.189 | -0.008 |  | -0.383 | -0.200 | -0.016 |  | -0.459 | -0.257 | -0.055 |  | -0.541 | -0.299 | -0.084 |
| LEG | 0.136 | 0.334 | 0.074 |  | -0.098 | 0.067 | 0.231 |  | 0.074 | 0.249 | 0.423 |  | 0.097 | 0.272 | 0.448 |  | 0.136 | 0.333 | 0.531 |  | 0.139 | 0.351 | 0.563 |
| SH | -0.398 | -0.164 | -0.337 |  | -0.276 | -0.092 | 0.092 |  | -0.337 | -0.126 | 0.084 |  | -0.338 | -0.124 | 0.091 |  | -0.398 | -0.163 | 0.070 |  | -0.412 | -0.161 | 0.089 |
| TH | -0.160 | 0.060 | -0.154 |  | -0.110 | 0.061 | 0.233 |  | -0.154 | 0.048 | 0.251 |  | -0.174 | 0.031 | 0.237 |  | -0.115 | 0.063 | 0.287 |  | -0.150 | 0.089 | 0.328 |
| E | -0.142 | 0.084 | -0.245 |  | -0.203 | 0.048 | 0.299 |  | -0.245 | 0.035 | 0.312 |  | -0.214 | 0.074 | 0.362 |  | -0.607 | -0.270 | 0.066 |  | -0.707 | -0.348 | 0.011 |
| E×SR(log-linear) | -0.036 | 0.082 | -0.098 |  | -0.100 | 0.032 | 0.163 |  | -0.098 | 0.048 | 0.193 |  | -0.010 | 0.053 | 0.204 |  | 0.072 | 0.104 | 0.280 |  | 0.067 | 0.122 | 0.310 |
| E×FG | -0.153 | -0.043 | -0.170 |  | -0.129 | -0.006 | 0.117 |  | -0.170 | -0.034 | 0.102 |  | -0.190 | -0.049 | 0.092 |  | -0.073 | 0.091 | 0.256 |  | -0.072 | 0.103 | 0.279 |
